# Mapping TB incidence across districts in Uganda to inform health program activities

**DOI:** 10.5588/ijtldopen.23.0624

**Published:** 2024-05-01

**Authors:** N.J. Henry, S. Zawedde-Muyanja, R.K. Majwala, S. Turyahabwe, R.V. Barnabas, R.C. Reiner, Jr, C.E. Moore, J.M. Ross

**Affiliations:** ^1^ Big Data Institute, Li Ka Shing Centre for Information Discovery, University of Oxford, Oxford, UK;; ^2^ Henry Spatial Analysis, Seattle, WA, USA;; ^3^ Infectious Diseases Institute, Makerere University, Kampala,; ^4^ Uganda Ministry of Health, National Tuberculosis and Leprosy Program, Kampala, Uganda;; ^5^ Division of Infectious Diseases, Massachusetts General Hospital, Boston, MA,; ^6^ Harvard Medical School, Cambridge, MA,; ^7^ Department of Health Metrics Sciences, University of Washington, Seattle, WA,; ^8^ Institute for Health Metrics and Evaluation, Seattle, WA, USA;; ^9^ The Centre for Neonatal and Paediatric Infection, Infection and Immunity Institute, St George’s, University of London, London, UK;; ^10^ Division of Allergy and Infectious Diseases, University of Washington, Seattle, WA, USA

**Keywords:** modelling, tuberculosis, TB prevention, TB control program

## Abstract

**BACKGROUND:**

Identifying spatial variation in TB burden can help national TB programs effectively allocate resources to reach and treat all people with TB. However, data limitations pose challenges for subnational TB burden estimation.

**METHODS:**

We developed a small-area modeling approach using geo-positioned prevalence survey data, case notifications, and geospatial covariates to simultaneously estimate spatial variation in TB incidence and case notification completeness across districts in Uganda from 2016–2019. TB incidence was estimated using 1) cluster-level data from the national 2014–2015 TB prevalence survey transformed to incidence, and 2) case notifications adjusted for geospatial covariates of health system access. The case notification completeness surface was fit jointly using observed case notifications and estimated incidence.

**RESULTS:**

Estimated pulmonary TB incidence among adults varied >10-fold across Ugandan districts in 2019. Case detection increased nationwide from 2016 to 2019, and the number of districts with case detection rates >70% quadrupled. District-level estimates of TB incidence were five times more precise than a model using TB prevalence survey data alone.

**CONCLUSION:**

A joint spatial modeling approach provides useful insights for TB program operation, outlining areas where TB incidence estimates are highest and health programs should concentrate their efforts. This approach can be applied in many countries with high TB burden.

## RÉSUMÉ

**CONTEXTE:**

L'identification des variations spatiales de la charge de morbidité de la TB peut aider les programmes nationaux de lutte contre la TB à allouer efficacement les ressources pour atteindre et traiter toutes les personnes atteintes de TB. Cependant, les limites des données posent des problèmes pour l'estimation de la charge de morbidité infranationale.

**MÉTHODES:**

Nous avons développé une approche de modélisation à petite échelle en utilisant des données d'enquête de prévalence géolocalisées, des notifications de cas et des covariables géospatiales pour estimer simultanément la variation spatiale de l'incidence de la TB et l'exhaustivité de la notification des cas dans les districts de l'Ouganda de 2016 à 2019. L'incidence de la TB a été estimée à l'aide 1) des données au niveau des grappes de l'enquête nationale sur la prévalence de la TB de 2014–2015, transformées en incidence, et 2) des notifications de cas ajustées pour tenir compte des covariables géospatiales de l'accès au système de santé. La surface de complétude des notifications de cas a été ajustée conjointement à l'aide des notifications de cas observés et de l'incidence estimée.

**RÉSULTATS:**

L'incidence estimée de la TB pulmonaire chez les adultes a été multipliée par >10 dans les districts ougandais en 2019. La détection des cas a augmenté à l'échelle nationale entre 2016 et 2019, et le nombre de districts avec des taux de détection des cas >70% a quadruplé. Les estimations de l'incidence de la TB au niveau des districts étaient cinq fois plus précises qu'un modèle utilisant uniquement les données de l'enquête sur la prévalence de la TB.

**CONCLUSION:**

Une approche conjointe de modélisation spatiale fournit des informations utiles pour le fonctionnement des programmes de lutte contre la TB, en décrivant les domaines où les estimations de l'incidence de la TB sont les plus élevées et où les programmes de santé devraient concentrer leurs efforts. Cette approche peut être appliquée dans de nombreux pays où la charge de morbidité de la TB est élevée.

Reducing the substantial burden of TB morbidity and mortality requires public health efforts that fit the local epidemiology of a region. While TB prevalence and incidence are typically estimated at the national level in high-burden settings, they likely vary locally within a country in relation to differences in underlying risk factors. By identifying these differences, programs can more efficiently and equitably allocate resources to reach and treat all people with TB to reduce the local burden of disease.^1,2^

In Uganda, the National Tuberculosis and Leprosy Control Program (NTLP) aims to provide effective and equitable treatment to all people with TB and provide TB preventive treatment to people with increased risk of developing TB. Uganda is a country with a high burden of TB, with an estimated incidence of 198/100,000 in 2022.^3^ Case notifications have increased by 59% over the past decade,^4^ thanks to campaigns to sensitize people about the need to seek care for TB symptoms, improved availability of rapid diagnostic testing for TB, and community screening by village health teams.

Despite recent increases in TB case notifications, a significant gap persists between the number of people estimated to have developed TB in 2021 and those who were notified to the NTLP through the national health information system.^5^ Previous studies suggest that TB burden varies widely across Uganda's districts and regions.^6–8^ Similarly, health and social factors associated with TB vary across the country: for example, HIV prevalence, a leading risk factor for TB, was found to vary four-fold across the regions of Uganda in 2020.^9^ Better estimation of subnational TB burden may help inform public health efforts to reach additional people with TB.

At the national level, TB burden has traditionally been measured using TB prevalence surveys, case notifications, and cause-specific mortality data, where available.^10,11^ National TB prevalence surveys use a well-defined screening process to identify people with signs and symptoms of TB within a sample population, typically people aged ≥15 years, and microbiological testing is conducted to confirm the presence of TB.^12^ National TB prevalence surveys, while providing robust epidemiological evidence, are conducted infrequently due to their expense and logistical complexity. In contrast, reported case notifications offer a more abundant and readily accessible source of data, with counts available for each geographic district and year. However, it is important to note that while case notifications provide valuable insight into the distribution of active TB diagnoses, they may not fully capture the true TB incidence due to various factors such as lack of access to healthcare, diagnostic limitations, or misdiagnosis as other respiratory conditions.^7^

Although previous studies have explored spatial variation in TB incidence and prevalence within high-burden countries, no widely accepted standards exist for assessing subnational variation in TB.^13^ A recent systematic review identified 168 studies that conducted spatial analyses of TB, including 57 in high-burden settings.^14^ Of these, 161 (96%) used TB case notifications as the data underlying the spatial analysis, although no studies in the review accounted for spatial variation in under-reporting of case notifications, which can be problematic in settings where patterns in case notifications may better reflect health system access and program efforts than the underlying TB burden.^15^ Additional factors such as distance from hospital/clinic, lack of local transport, gender, knowledge of TB, and stigma can all influence treatment-seeking behavior,^16–18^ further complicating the interpretation of case notification rates. Subsequent spatial studies have used TB prevalence survey data to estimate subnational burden.^13,19^

This study proposes a new method for estimating spatial variation in TB burden by combining data from a TB prevalence survey, annual case notifications, and predictive spatial covariates. This method is applied to estimate TB incidence and case notification completeness (defined as the estimated proportion of incident pulmonary TB cases among adults aged ≥15 years who are reported in case notifications) across districts in Uganda, with the goal to inform public health program activities to reach all people with TB.

## METHODS

### Study design and ethics

This nationwide modeling study used the 136 administrative districts of Uganda as the unit of analysis, including the capital, Kampala, as a separate administrative unit.

The study was approved by the Makerere University School of Medicine Research Ethics Committee, Kampala, Uganda (2021-019); the Uganda National Council for Science and Technology, Kampala, Uganda (HS1981ES); and the University of Washington Human Subjects Division, Seattle, WA, USA (STUDY00006169).

### Data sources

**Figure 1.**
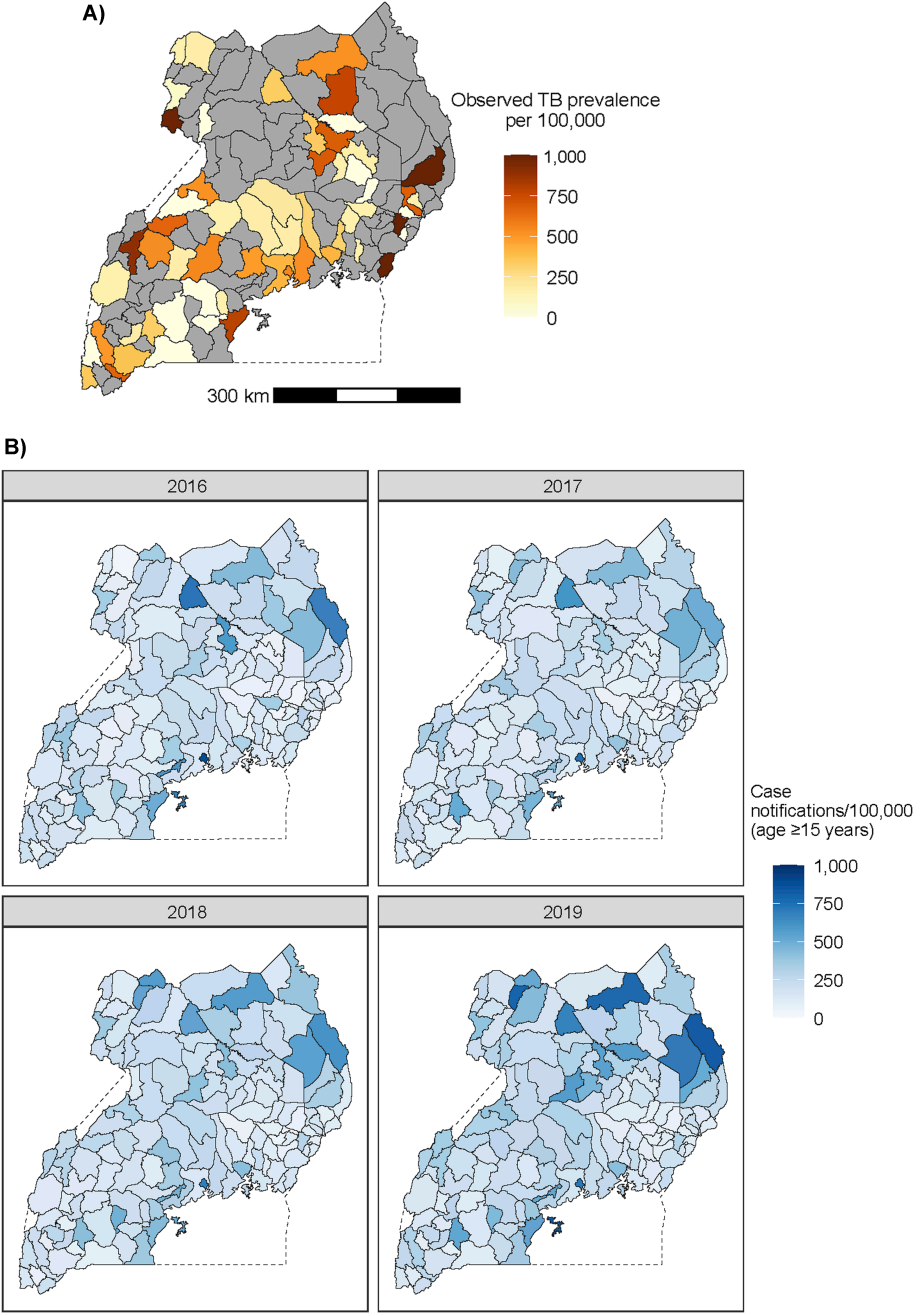
Data sources used to estimate TB incidence and case notification completeness: **A)** TB prevalence point estimates from the 2014–2015 National Tuberculosis Prevalence Survey, aggregated to the district level; **B)** TB case notification rates by district, 2016–2019.

The 2014–2015 Uganda National TB Prevalence Survey used a cross-sectional, population-based cluster sampling design.^20^ We matched each sampled survey cluster to its corresponding district. We extracted the number of people with bacteriologically confirmed pulmonary TB and the total tested population within each cluster from the National TB Prevalence Survey published report.^20^ Figure 1A shows these raw prevalence estimates by district. Of 136 districts in Uganda, 58 were associated with at least one cluster from the 2014–2015 prevalence survey.

We extracted annual counts of TB case notifications by district, as reported to the NTLP, for 2016–2019. We included only case notifications for pulmonary TB among adults aged ≥15 years to match the population in the 2014–2015 National TB Prevalence Survey. We then calculated the population of adults aged ≥15 years using high-resolution gridded population estimates for every 1 km-by-1 km area in Uganda from the WorldPop Project.^21^ We aggregated these data by district. Figure 1B shows the estimated case notification rate for pulmonary TB by district. At the national level, the case notification rate for pulmonary TB among people aged 15 and above increased from 211/100,000 people in 2016 to 254/100,000 in 2019.^5^

Through consultation with experts in Uganda, we identified five predictive covariates for TB incidence, all of which vary by district and year: 1) household crowding;^22^ 2) nighttime lights, a proxy for local variation in economic activity;^23^ 3) HIV prevalence;^24^ 4) refugees per capita;^25^ and 5) cattle per capita, a proxy for pastoral populations.^26^ We also identified one predictive covariate for TB case notification reporting completeness, which varies by district: average travel time to the nearest health facility.^27^ All covariates were summarized by district and year for use in the statistical model.

### Statistical model

**Figure 2.**
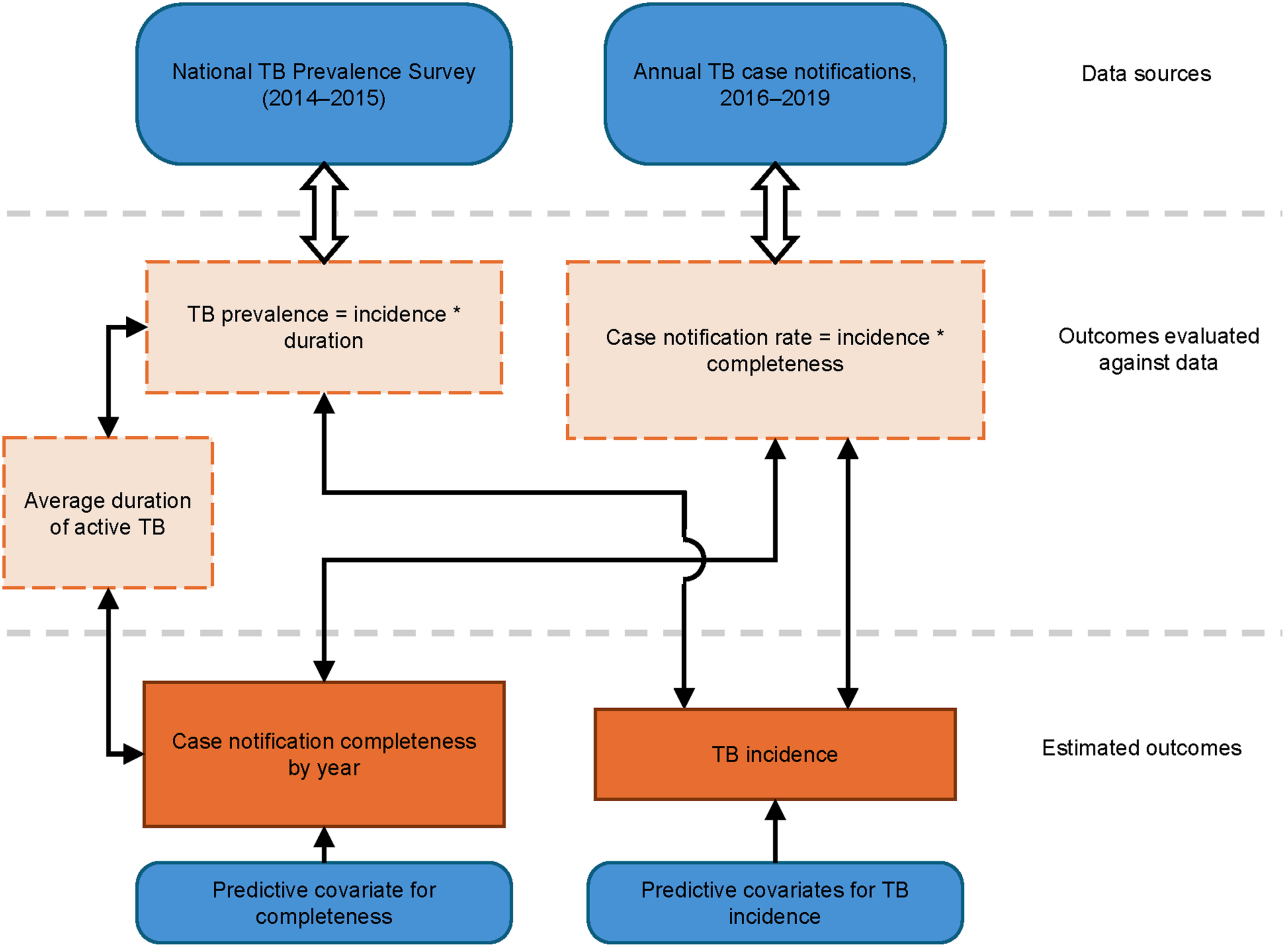
Flow chart for small area estimation model. TB incidence is estimated by district, and case notification completeness is estimated by district and year. Blue boxes indicate data inputs to the model. Dark orange boxes indicate the two key outcomes, TB incidence and case notification completeness, estimated by the model. Light orange boxes indicate intermediate outcomes that are used to compare estimated outcomes to data.

We developed a small-area statistical model to jointly and simultaneously estimate TB incidence and TB case notification reporting completeness by district for 2016-2019, summarized in Figure 2. TB incidence for each district *d* and year *t*, denoted *Incidence*_*d*,*t*_, is a log-linear surface that varies according to an intercept (*α*^*INC*^), fixed effects (${\vec{\beta }}^{\text{INC}}$) on the five incidence covariates ($X_{d,t}^{INC}$, a matrix), and a spatially-structured random intercept by district (${\vec{Z}}_{d}^{INC}$):

$${Incidence}_{d,t}=\exp\left(\alpha^{INC}+{\vec{\beta }}^{\text{INC}}X_{d,t}^{INC}+{\vec{Z}}_{d}^{INC}\right)$$

We define case notification reporting completeness as the estimated ratio between the number of reported TB cases and true count of incident TB cases as defined in our study. Completeness for each district *d* and year *t*, denoted *Completeness*_*d*,*t*_, is a logit-linear surface that varies according to an intercept (*α*^*COMP*^), a fixed effect (*β*^*COMP*^) on the distance to facility covariate ($X_{d}^{COMP}$, a column matrix), a spatially-structured random intercept (${\vec{Z1}}_{d}^{COMP}$), and a spatially-structured random slope on time (${\vec{Z2}}_{d}^{COMP}$):

$${Completeness}_{d,t}=logit^{-1}\left(\alpha^{COMP}+\beta^{COMP}X_{d}^{COMP}+{\vec{Z1}}_{d}^{COMP}+{\vec{Z2}}_{d}^{COMP}*t\right)$$

The two spatial random intercepts (${\vec{Z}}_{d}^{INC},{\vec{Z1}}_{d}^{COMP}$) and spatial random slope on time (${\vec{Z2}}_{d}^{COMP}$) are all parameterized using a Besag proper conditional autoregressive (CAR) model in space.^28^

In populations where disease burden is relatively stable, the prevalence and incidence of a disease are related by the expected (mean) duration of the disease: in other words, *Prevalence* = Incidence*Duration.^29^ To relate TB prevalence data to estimates of TB incidence, it is therefore necessary to estimate TB duration by district. Using TB duration estimates published by the WHO that vary depending on treatment and HIV status,^10^ we develop a formula for average duration as a function of case notification completeness, detailed in the Supplementary Appendix:

$${Duration}_{d,t}=1.51\;years\;-\;0.42*Completeness_{d,t}$$

District-level observations from the National TB Prevalence Survey, with numerators $Y_{d}^{Prev}$ and sample sizes $N_{d}^{Prev}$, are evaluated against the estimated prevalence (incidence times duration) surface in 2016, which is the earliest year where district-level case notifications were readily available:

$$Y_{d}^{PREV}∼Poisson\left(N_{d}^{PREV}*{Incidence}_{d,2016}*{Duration}_{d,2016}\right)$$

In addition, case notifications with reported cases $Y_{d,t}^{Notif}$ and corresponding population denominators $N_{d,t}^{Notif}$ are evaluated against the true incident cases in the population ($N_{d,t}^{Notif}*\;Incidence_{d,t}$) multiplied by case notification completeness:

$$Y_{d,t}^{Notif}∼Poisson\left(N_{d,t}^{Notif}*\;Incidence_{d,t}*Completeness_{d,t}\right)$$

The joint model was fit using the Laplace approximation for mixed-effect parameter estimation.^30^ The model was fit in R v.4.3.1 (R Core Team, Vienna, Austria) using the package Template Model Builder v.1.9.6.^30,31^ The likelihood of all model parameters governing district-level TB incidence and case notification completeness were evaluated simultaneously, then repeatedly stepped until reaching the most likely combination of parameters given the observed data and model priors.

### Model evaluation and comparison

To understand the effect of case notifications on model performance, we fit two versions of the model: one incorporating both TB prevalence survey results as well as case notifications, and a prevalence-only small area model incorporating just data from the TB prevalence survey.

We performed out-of-sample predictive validity testing using the TB Prevalence Survey. We also performed sensitivity analyses to understand how assumptions about average duration influenced model results. These tests are detailed in the Supplementary Data.

## RESULTS

**Figure 3.**
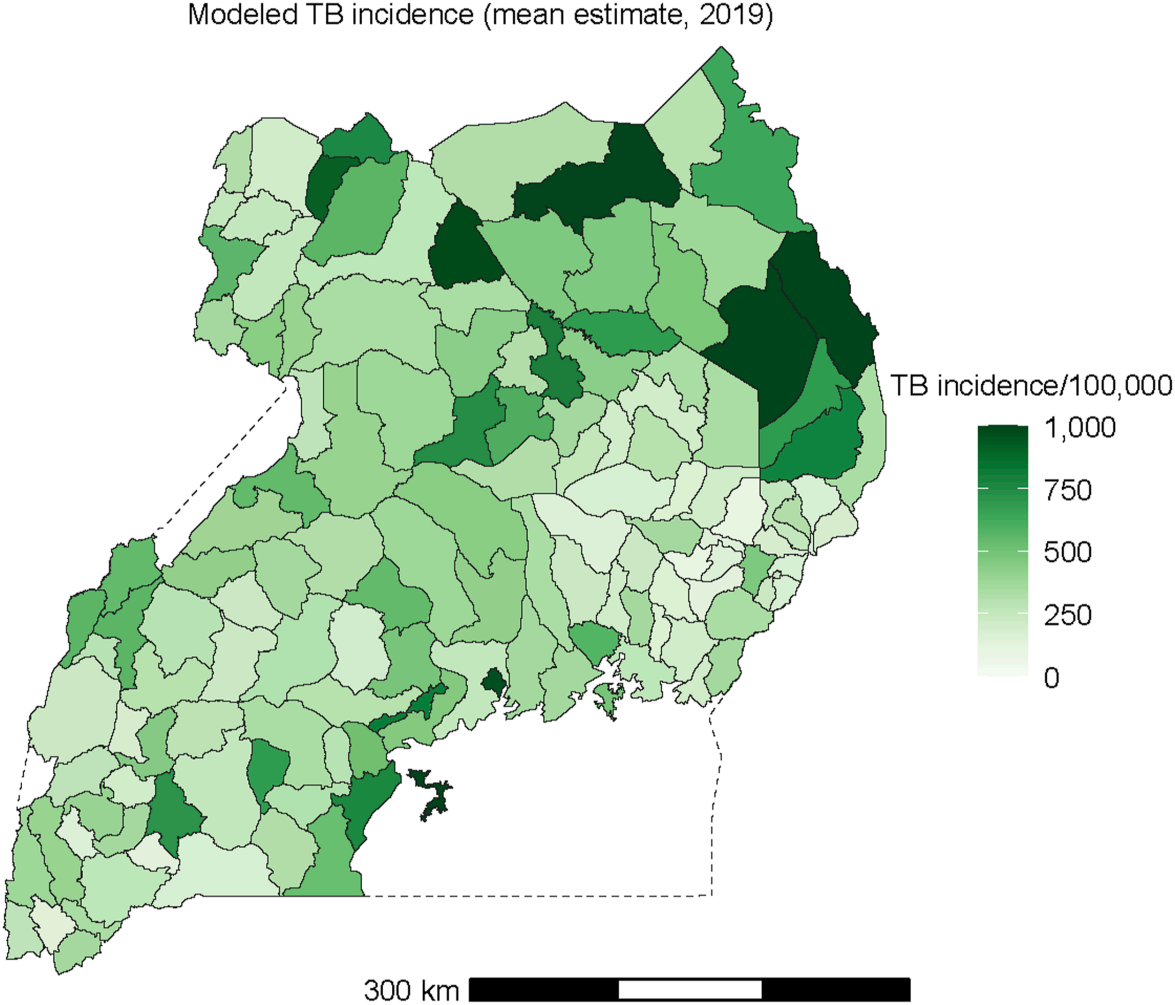
Estimated incidence of pulmonary TB/100,000 population by district in Uganda, 2019.

Figure 3 shows the estimated incidence of pulmonary TB across the districts of Uganda. The joint spatial model estimated that TB incidence varied over 10-fold across districts of Uganda, ranging from 94 cases/100,000 in Bukedea District, Eastern Region to 1,313 cases/100,000 in Kalangala District, Central Region. District clusters with below-average TB incidence were apparent in the southwest and southeast of Uganda, while districts with above-average TB incidence were concentrated in the center and north of the country.

**Figure 4.**
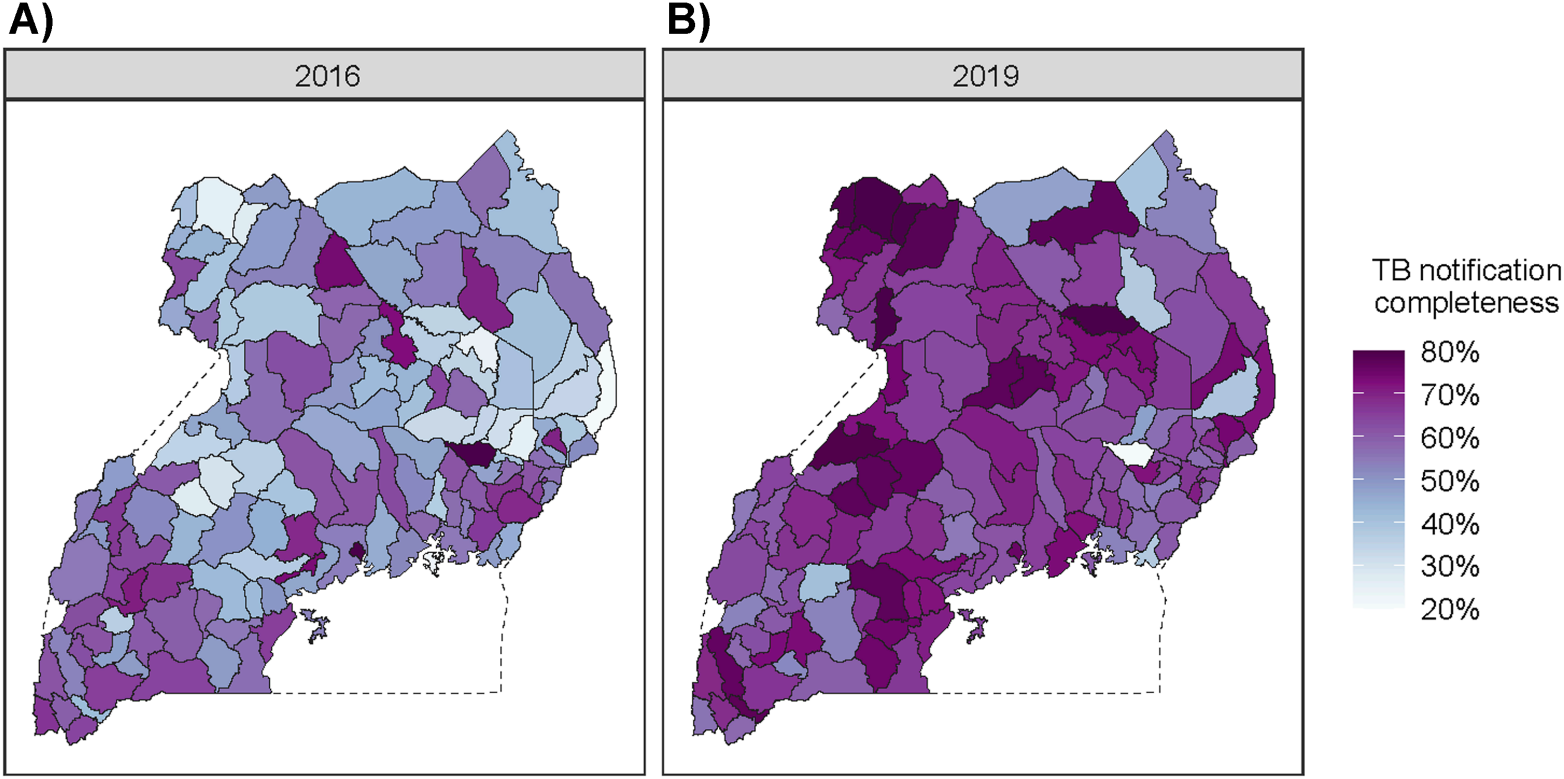
Estimated TB case detection rate by district in Uganda for **A)** 2016 and **B)** 2019.

Figure 4 shows the estimated case detection rate (defined as the ratio between observed case notification counts and model-estimated TB incidence) by district in 2016 and 2019. Case detection increased in 109 of 136 districts during the study time period. In 2016, fewer than 1 in 10 districts had a case detection rate greater than 70%, while 4 in 10 districts had a case detection rate below 50%. By 2019, over 3 in 10 districts had case detection greater than 70%, and fewer than 1 in 10 districts had case detection rates below 50%. This matches evidence from the NTLP, which recorded a 33% increase in case notifications from 2016 to 2019. However, the model estimates that some clusters of low case detection remain in districts across the far north and east of Uganda.

The average duration of TB cases was also estimated as a function of the case detection rate. In 2019, the average duration of pulmonary TB was estimated to range from 1.15 years in Obongi to 1.43 years in Pallisa, a difference of over 3 months.

### Results of model comparison

A second, survey-only model was developed for comparison using only observations from the 2014–2015 national TB prevalence survey and the same suite of five predictive covariates (i.e., without case notification data). Compared to the data shown in Figure 1, the survey-only model strongly smooths towards the national mean in unobserved districts. Prevalence estimates generated from the survey-only model are also much more uncertain than the joint model, with the average width of the 95% uncertainty interval (UI) more than five times larger.

**Figure 5.**
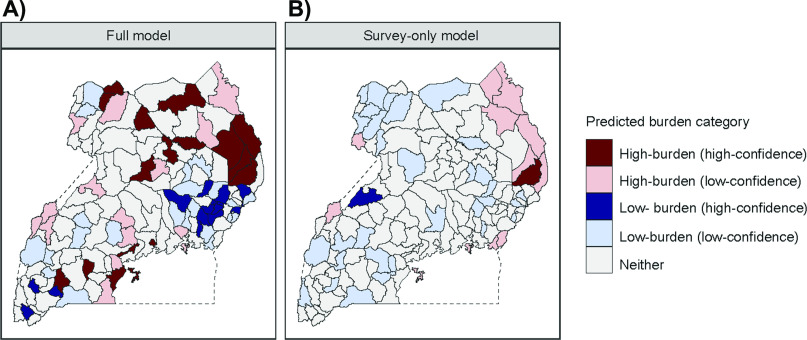
Results of a performance comparison between the statistical model presented in **A)** this manuscript, and **B)** an alternative small-area model that does not incorporate data from TB case notifications.

Figure 5 demonstrates how the addition of case notification data increases model precision, enabling greater confidence in the identification of low- and high-burden districts. The figure shows model predictions for districts where TB prevalence falls below 300 cases/100,000, as well as districts exceeding a relatively high prevalence threshold of 600/100,000. In the context of this figure, low-confidence predictions indicate that the model's mean estimated prevalence passed a given threshold, while high-confidence predictions indicate that both bounds of the 95% uncertainty interval for prevalence in a district have passed the threshold. Figure 5A shows prevalence estimates based on the joint model developed in this paper; Figure 5B shows prevalence estimates based on the survey-only model. The joint model sorted half of all districts (*n* = 68) into either low or high burden categories; it sorted 31 districts with high confidence. The survey-only model sorted only 45 of 122 districts into either high or low burden categories, and sorted only three districts with high confidence.

When comparing out-of-sample results between the two models, the joint model outperformed the survey-only model, displaying a lower root mean squared error and a higher correlation to held-out prevalence survey data (Supplementary Figure S5). Out-of-sample estimates from the joint model were the most consistent with both prevalence survey data and observed case notifications.

## DISCUSSION

We describe a novel framework for estimating TB incidence and case detection rate per district by synthesizing data from a national TB prevalence survey and annual case notifications in Uganda. We found that although estimated incidence varied substantially by district, the estimated case detection rate increased between 2016 and 2019 in most districts.

More precise estimates of high vs. low burden districts can be informative for public health program activities. In many settings, district TB case-finding targets are developed by multiplying estimated national TB incidence by the district-level population; alternately, case-finding targets by district may be set as a function of the previous year’s targets. By incorporating district-level estimates of TB incidence into case-finding targets, TB programs may be better able to identify all people living with TB in a district. District specific estimates generated by the model were shared with Uganda NTLP and have been used to guide active TB case finding campaigns.

This modeling approach that uses prevalence survey and case notification data together generates more precise estimates by compensating for the shortcomings of each data source, particularly by addressing the issue of systematic under-reporting in case notification data. Modeling approaches utilizing case notification data are valuable, particularly as the quality of these data improve through major investments in electronic health management information systems and efforts to improve outreach to find people with TB. This approach represents a step forward from previous TB spatial modeling frameworks, which have relied solely on one of these two data types.^14^

While this model provides relatively precise estimates of TB prevalence and case notification completeness across districts in Uganda, it is important to acknowledge its limitations. First, this model assumes that changes in incidence can be explained by covariate relationships, and that the random intercept on TB prevalence (a stand-in for latent factors causing variation across districts) remains constant over time. While this assumption may be reasonable over the study time period,^2,11^ it cannot hold if the End TB goals are to be met. Additional population-based surveys of TB burden are needed to ground future estimates as the relationship between burden and underlying risk factors shifts over time, particularly following major disruptions in TB programming due to COVID-19. Second, to relate data sources measuring TB incidence and prevalence, we approximate variation in TB duration by district as a function of case detection rates. This approximation required strong assumptions about the factors influencing duration by district in Uganda. More evidence is needed to understand local variation in TB duration. Because subnational TB modeling relies on statistical assumptions, results should be interpreted in conjunction with local epidemiological evidence and expertise.

While this study focused on Uganda, similar methods could be applied in other high-burden countries with TB prevalence survey data, which includes at least 23 of the 30 high TB burden countries.^2^ By modeling TB case notifications with data from a single prevalence survey, programs in many TB high-incidence countries could utilize information about subnational TB variation to reach and treat more people with TB, potentially reducing the burden of TB.

## Supplementary Material


