# Supplementary material for: Mapping TB incidence across districts in Uganda to inform health program activities
Source: IJTLD Open. 2024 May 1;1(5):223–9. doi: 10.5588/ijtldopen.23.0624 (PMC11249603; doi:10.5588/ijtldopen.23.0624)
Supplement: Supplementary file 1 [file iutld_ijtld_open_23.0624_supplementarydata1.pdf]

http://dx.doi.org/10.5588/ijtldopen.23.0624

SUPPLEMENTARY DATA

Mapping TB incidence across districts in Uganda to inform health program activities

Henry, N. J., Zawedde-Muyanja, S., Majwala, R. K., Turyahabwe, S., Barnabas, R. V., Reiner, R. C., Moore, C. E., Ross, J. M. (2024). Mapping TB incidence across districts in Uganda to inform health program activities. *IJTLD Open*.

Contents

I. Estimating duration as a function of case notification completeness ... 3
II. Model hyperparameters and priors ... 6
III. Model covariates ... 7
A. Covariate preparation ... 7
B. Fitted covariate fixed effect coefficients ... 7
IV. Sensitivity analysis using different duration assumptions ... 8
A. Lower TB-HIV coinfection rate ... 8
B. Fixed duration of 2.15 years ... 9
C. Fixed duration of 1 year ... 11
V. Out-of-sample comparison to a prevalence-only model ... 13
A. Validation against prevalence survey data ... 13
B. Comparison to a prevalence floor ... 14
C. Validation against an adjusted prevalence dataset ... 16
VI. References ... 18

Supplementary Figures and Tables

Supp. Figure 1: Estimated incidence of pulmonary TB per 100,000 population by district in Uganda, 2019, using an alternative model based on a lower TB-HIV coinfection rate ... 8

Supp. Figure 2: Scatterplot comparing estimated TB incidence in 2019 between the default model described in the manuscript (X axis) and an alternative model based on a lower TB-HIV coinfection rate (Y axis). 95% uncertainty intervals for both sets of estimates are shown as horizontal and vertical bars. .. 9

Supp. Figure 3: Estimated incidence of pulmonary TB per 100,000 population by district in Uganda, 2019, using an alternative model with a fixed duration of 2.15 years ... 10

Supp. Figure 4: Scatterplot comparing estimated TB incidence in 2019 between the default model described in the manuscript (X axis) and an alternative model with a fixed duration of 2.15 years (Y axis). 95% uncertainty intervals for both sets of estimates are shown as horizontal and vertical bars ... 10

Supp. Figure 5: Estimated incidence of pulmonary TB per 100,000 population by district in Uganda, 2019, using an alternative model with a fixed duration of 1 year 11

Supp. Figure 6: Scatterplot comparing estimated TB incidence in 2019 between the default model described in the manuscript (X axis) and an alternative model with a fixed duration of 1 year (Y axis). 95% uncertainty intervals for both sets of estimates are shown as horizontal and vertical bars. 12

Supp. Figure 7: Scatterplots comparing observed district-level prevalence from the 2014-2015 National TB Prevalence Survey (X axis) with out-of-sample predictions for TB prevalence in those districts (Y axis) for the three candidate models. The X=Y line is shown as a gray dashed line, representing the trend if the out-of-sample models perfectly predicted all prevalence survey data........ 14

Supp. Figure 8: Scatterplots comparing out-of-sample prevalence predictions from the two spatial models (Y axis) against a “prevalence floor” calculated from case notification rates (X axis). The Y=X line is shown as a gray dashed line; estimates falling below this are inconsistent with observed case notifications. 15

Supp. Figure 9: Scatterplot comparing district-level prevalence estimates from the National TB Prevalence Survey, on the X axis, with the minimum prevalence that would be consistent with observed case notifications, on the Y axis. The original data are shown as solid blue points; the adjusted validation data points are shown in the black point outlines........ 16

Supp. Table 1: Plausible duration of clinically active tuberculosis by HIV coinfection and treatment status, as published alongside the WHO Global Tuberculosis Report 2023 3

Supp. Table 2: Select parameters in the joint TB model and associated priors. 6

Supp. Table 3: Fitted fixed effect coefficients on model covariates. The mean estimate and 95% uncertainty interval bounds are provided for each fixed effect........ 7

Supp. Table 4: In-sample and out-of-sample predictive metrics for the three model alternatives when comparing to unadjusted TB Prevalence Survey data, aggregated by district. RMSE and UI width are both defined in units of prevalent cases per 100,000 population. 13

Supp. Table 5: In-sample and out-of-sample predictive metrics for the three candidate models when comparing to the adjusted validation dataset. RMSE and UI width are both defined in units of prevalent cases per 100,000 population........ 16

I. Estimating duration as a function of case notification completeness

In populations where disease burden is relatively stable, the prevalence and incidence of a disease are related by the expected (mean) duration of the disease: $Prevalence = Incidence * E[Duration]$.[1] When the distribution of active TB durations across a population are relatively stable, TB duration by district could then be used to crosswalk between data sources designed to measure TB prevalence and incidence.

The mean duration of tuberculosis in a population can vary based on factors such as HIV prevalence, rates of case detection and treatment, and delays between the onset of active TB and the beginning of treatment. If a person with TB is immediately treated, the preferred treatment strategy for drug-susceptible TB includes 26 weeks, or approximately 6 months, of chemotherapy.[2] At the upper range of duration, natural history studies suggest that the mean duration of untreated pulmonary TB among HIV negative adults is approximately three years.[3] The average duration of clinically active TB among adults with HIV is likely to vary based on antiretroviral therapy (ART), possible interactions between drugs used to treat TB and HIV, and higher mortality among individuals coinfected with HIV-TB.[3,4] Treatment duration is longer for people with drug-resistant TB, although the recommended treatment regimen has recently been shortened to 9-12 months for people without a prior history of TB treatment, compared to historical courses of nearly two years.[5,6] A reasonable estimate for the expected duration of active TB in a population should combine the experiences of untreated and treated individuals, accounting for HIV coinfection and possible treatment delay, and should fall between the extremes of 6 months and three years.

The WHO TB group estimates plausible duration ranges of TB disease by HIV and treatment status, then estimates duration at the national level as a population-weighted combination of these groupings.[7] These groupings, plausible duration distributions for individuals within each grouping, and expected duration across each grouping are shown in Supp. Table 1, below. The expectation of duration across each grouping is calculated as $E[Uniform(a,b)] = (a+b)/2$.

Supp. Table 1: Plausible duration of clinically active tuberculosis by HIV coinfection and treatment status, as published alongside the WHO Global Tuberculosis Report 2023

Group	Case category	Distribution of duration, in years	***E[duration]*, in years**
a	Treated, HIV negative	Uniform(0.2, 2)	1.100
b	Not treated, HIV negative	Uniform(1, 4)	2.500
c	Treated, HIV positive	Uniform(0.01, 1)	0.505
d	Not treated, HIV positive	Uniform(0.01, 0.2)	0.105

We apply the WHO approach to estimate TB duration by Ugandan district based on expected rates of TB-HIV coinfection and TB treatment. The formula we apply to estimate expected duration in each district is a weighted average across the four groups identified in Supp. Table 1, adjusting for treatment delay:

$$E[D_{total}] = (E[D_a] + L_a)Prop_a + E[D_b]Prop_b + (E[D_c] + L_c)Prop_c + E[D_d]Prop_d$$

In this equation, $E[D_{<a,b,c,d>}]$ represents the expected (average) duration within each WHO grouping specified in Supp. Table 1, while $Prop_{<a,b,c,d>}$ represents the proportion of individuals with active TB that fall within each grouping. The variables L_a and L_c denote the average delay between the onset of

active TB and the beginning of treatment for HIV negative (a) and HIV positive (c) adults who receive treatment. Based on a 2014 study of two districts in Uganda which estimated an average treatment delay of four weeks (0.077 years),[8] we set $L_a = L_c = 0.077$.

While Dwyer-Lindgren *et al.* estimated that HIV prevalence among adults varied from 1.7% to 11.5% across the districts of Uganda as of 2017,[9] the relationship between HIV prevalence, TB prevalence, and HIV-TB coinfection at the subnational level is not well-described. The Global Burden of Disease Study estimates that 41.3% of all people with prevalent TB in Uganda were also living with HIV in 2017.[10] This aligns with the proportion of adults diagnosed with TB in Uganda who also have HIV, as reported by case notifications: as of 2017-2018, the Uganda NTLP reported that the TB/HIV coinfection rate was 40% based on case notifications data.[11] However, the 2014-2015 National TB Prevalence Survey in Uganda estimated just 27% adults with active TB also had HIV.[12] A WHO modeling study observed this same discrepancy, with case notifications reporting higher TB/HIV coinfection rates than prevalence surveys, across seven countries with high TB and HIV prevalence.[13] Likely reasons for this discrepancy include that people living with HIV may access care and be screened for TB more frequently than people without HIV, and may have a shorter TB illness duration because of higher mortality.[14]

Given uncertainty around district-level variation in HIV-TB coinfection and treatment rates across Uganda, this study makes simplifying assumptions to estimate $Prop_{<a,b,c,d>}$ as a function of TB case notification reporting. We assume the nationwide TB/HIV coinfection rate to be 41.3% based on Global Burden of Disease estimates, which also align with findings from case notifications;[10] we apply this ratio uniformly across the country to estimate the relative prevalence of HIV positive and HIV negative TB cases by district. Proportions of treated versus untreated cases area estimated by multiplying the TB case detection rate by the treatment success rate, which we calculated to be 71% nationwide in the Uganda NTLP annual report from 2017-18, the midpoint of estimated time series.[11] Finally, treatment rates were assumed to be constant for people with TB among individuals with and without HIV, so that $Prop_a = (Prop_{HIV} * Prop_{Treat})$, and so on. These assumptions allow for the estimation of the proportion of all prevalent pulmonary TB cases that fall into each WHO category based on the case detection rate, denoted as π:

$$Prop_a = Prop_{Treat} * (1 - Prop_{HIV}) = .71\pi * .587 = .42\pi$$

$$Prop_b = (1 - Prop_{Treat}) * (1 - Prop_{HIV}) = (1 - .71\pi) * .587 = .587 - .42\pi$$

$$Prop_c = Prop_{Treat} * Prop_{HIV} = .71\pi * .413 = .29\pi$$

$$Prop_d = (1 - Prop_{Treat}) * Prop_{HIV} = (1 - .71\pi) * .413 = .413 - .29\pi$$

$$Prop_a + Prop_b + Prop_c + Prop_d = 1$$

Using these simplifying assumptions, the average duration of pulmonary TB in a district can be estimated as a function of the case reporting completeness π:

$$D(\pi) = E[D_{total}]$$

$$= (E[D_a] + L_a)Prop_a + E[D_b]Prop_b + (E[D_c] + L_c)Prop_c + E[D_d]Prop_d$$

$$= (1.1 + .077)(.42\pi) + 2.5(.587 - .42\pi) + (.505 + .077)(.29\pi) + 0.105(.413 - .29\pi)$$

$$= 1.51 - .42\pi$$

This formula is used in the joint TB model to describe the average duration of TB by district, and therefore the relationship between TB incidence and prevalence, as a function of TB case notification completeness.

II. Model hyperparameters and priors

The joint spatial model was fit in an empirical Bayesian framework, with hyperparameters governing the relationships between individual model effects. These hyperparameters and corresponding priors are described in Supp. Table 2, below. This table can be cross-referenced against the model terms described in the manuscript's *Statistical Model* section. Note that the exponential priors on the σ terms can be interpreted as Penalized Complexity priors, as described by Simpson *et al.*[15]

<u>Supp. Table 2</u>: Select parameters in the joint TB model and associated priors.

Parameters used to estimate TB incidence (log space)		
**Model terms**	**Interpretation**	**Prior**
$\vec{\beta}^{INC}$	Fixed effect coefficients on the covariates for TB incidence, excluding the intercept	$N(0, 3^2)$ on each
$\rho_{\vec{Z}^{INC}}$	Hyperparameter: spatial autocorrelation between districts in the spatially-structured random intercept on TB incidence	$Beta(2.5, 1.5)$
$\sigma_{\vec{Z}^{INC}}$	Hyperparameter: standard deviation of the spatially-structured random intercept on TB incidence	$Exponential(scale = 1)$
Parameters used to estimate TB case notification completeness (logit space)		
**Model terms**	**Interpretation**	**Prior**
β^{COMP}	Fixed effect coefficient on the covariate for case notification completeness, excluding the intercept	$N(0, 3^2)$
$\rho_{\overrightarrow{Z1}^{COMP}}$	Hyperparameter: spatial autocorrelation between districts in the spatially-structured random intercept on case notification completeness	$Beta(2.5, 1.5)$
$\rho_{\overrightarrow{Z2}^{COMP}}$	Hyperparameter: spatial autocorrelation between districts in the spatially-structured random time slope on case notification completeness	$Beta(2.5, 1.5)$
$\sigma_{\overrightarrow{Z1}^{COMP}}$	Hyperparameter: standard deviation of the spatially-structured random intercept on case notification completeness	$Exponential(scale = 1)$
$\sigma_{\overrightarrow{Z2}^{COMP}}$	Hyperparameter: standard deviation of the spatially-structured random time slope on case notification completeness	$Exponential(scale = 1)$

III. Model covariates

A. Covariate preparation

Data sources for all covariates are cited in the Methods section of the manuscript. All covariates were available as high-resolution gridded raster surfaces, except for refugees per capita, which was estimated by district. Gridded raster covariates were aggregated to the district level by taking the population-weighted mean across pixels in the district, using gridded raster population estimates from the WorldPop project.[16] Nighttime light brightness measurements are commonly log-transformed before making comparisons to economic activity;[17] following this practice, we log-transformed the nighttime light brightness measurements by district. We then rescaled all covariates to have a mean equal to 0 and a standard deviation equal to 1.

We performed a Variance Inflation Factor (VIF) test for multi-collinearity on the covariates prior to the regression analysis.[18] None of the covariates exceeded a VIF threshold of 5, and so all candidate predictors were included in the final model.

B. Fitted covariate fixed effect coefficients

Because each covariate was rescaled before inclusion in the model, fixed effects have a consistent interpretation across covariates: the magnitude of each fixed effect describes the marginal effect of one standard deviation increase in the covariate on the outcome, in transformed space (log space for TB prevalence, log-odds space for case notification completeness).

Supp. Table 3 lists fitted fixed effect coefficients for all model covariates. These effect sizes should be interpreted with caution: fixed effect coefficients are conditional on all other effects, including the spatially-structured model terms. Also, due to the modifiable areal unit problem, a regression run on a different spatial scale might find different associations between these covariates and the outcomes.

Supp. Table 3: Fitted fixed effect coefficients on model covariates. The mean estimate and 95% uncertainty interval bounds are provided for each fixed effect.

Covariate name	Covariate on	Fixed effect coefficient
Household crowding	TB incidence	0.0585 (-0.0477 to 0.1799)
Nighttime light brightness, log-transformed	TB incidence	0.1207 (0.0162 to 0.2279)
HIV prevalence	TB incidence	0.1027 (0.0122 to 0.2029)
Refugees per capita	TB incidence	-0.0004 (-0.0962 to 0.0857)
Cattle per capita	TB incidence	0.0321 (-0.0729 to 0.1447)
Travel time to a healthcare facility, motorized	Case notification completeness	-0.1450 (-0.2596 to -0.0201)

IV. Sensitivity analysis using different duration assumptions

We conducted sensitivity analyses to test the effect of different duration assumptions on the joint model results. This section presents the results of these sensitivity analyses.

A. Lower TB-HIV coinfection rate

For the duration calculation, we assumed that the 41.3% of people aged 15 and above with active pulmonary TB also have HIV, which corresponds to estimates from the Global Burden of Disease 2019 study as well as reported TB-HIV coinfection rates from case notifications.[10,11] However, the 2014-2015 National TB Prevalence Survey found that 27% of survey participants aged 15 and above with active pulmonary TB were coinfected with HIV. For this sensitivity analysis, we update the value of $Prop_{HIV}$ from .413 to .27 in the expected duration calculation above, yielding a new formula for duration as a function of case notification completeness π:

$$D(\pi) = 1.83 - .59\pi$$

The results of this alternate model are shown below in Supp. Figure 1, using the same color scheme as manuscript Figure 3. The alternate model estimated that TB incidence ranged from a low of 86 cases per 100,000 in Bukedea District, Eastern Region to a high of 1,253 cases per 100,000 in Kalangala District, Central Region. The 2019 incidence estimates from alternative model are compared with the manuscript estimates in Supp. Figure 4. Compared to the base model, the alternate model yielded mean incidence estimates that were 7.6% lower on average, but none of these differences were statistically significant.

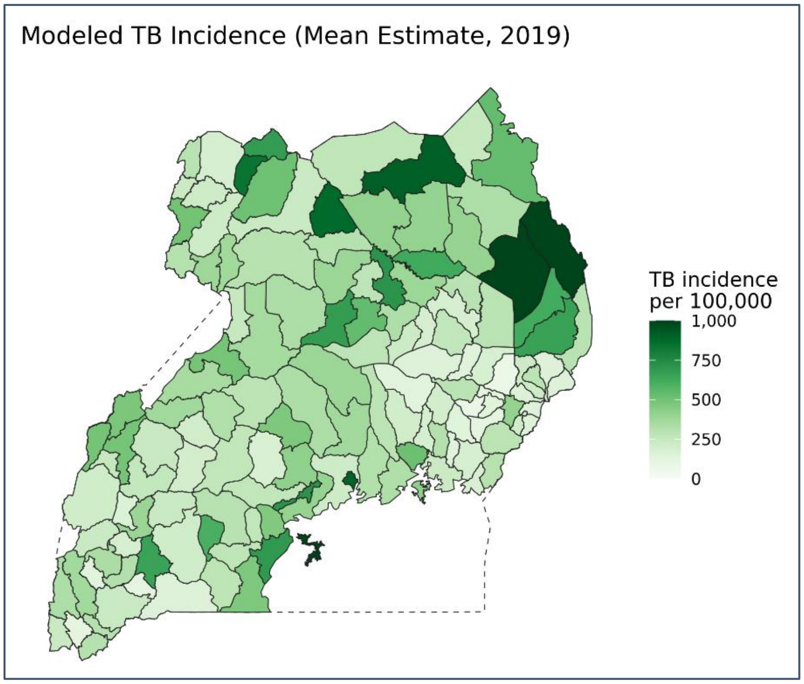

Supp. Figure 1: Estimated incidence of pulmonary TB per 100,000 population by district in Uganda, 2019, using an alternative model based on a lower TB-HIV coinfection rate

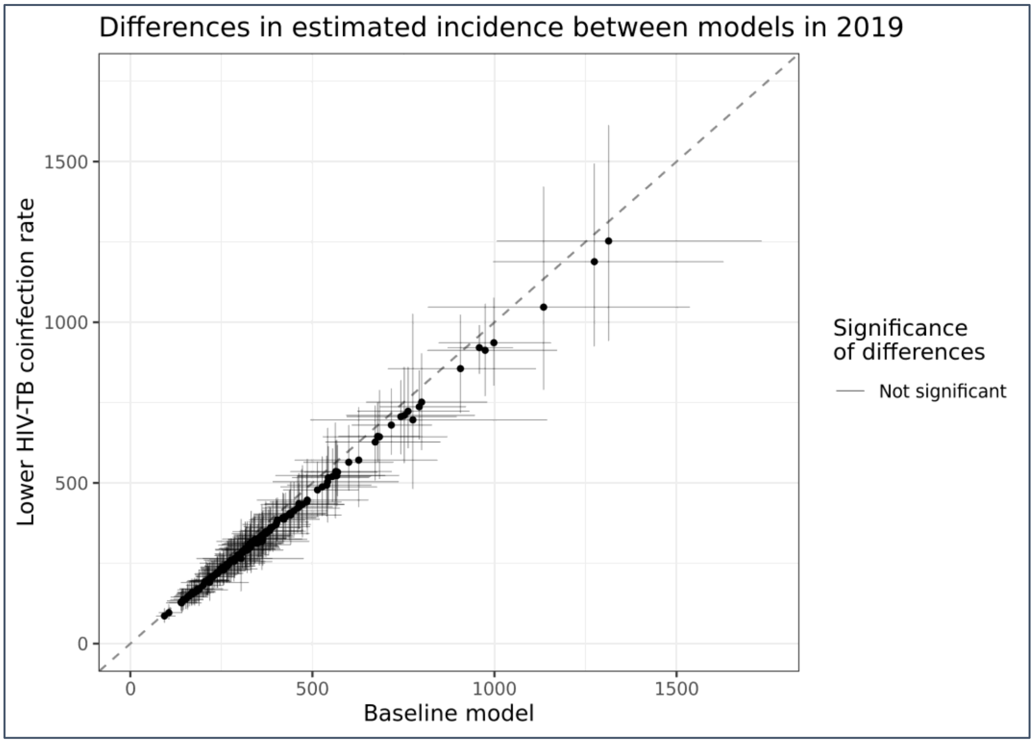

Supp. Figure 2: Scatterplot comparing estimated TB incidence in 2019 between the default model described in the manuscript (X axis) and an alternative model based on a lower TB-HIV coinfection rate (Y axis). 95% uncertainty intervals for both sets of estimates are shown as horizontal and vertical bars.

B. Fixed duration of 2.15 years

Our approach builds on the WHO method for estimating duration of active TB in a population as a combination of expected durations for sub-groups with differing HIV coinfection and treatment status. However, the Institute for Health Metrics and Evaluation (IHME) uses a different approach, estimating duration of active TB based on a Health Access and Quality (HAQ) Index.[19] Although the HAQ index is not available by Ugandan district, we conducted a sensitivity analysis using a fixed duration of 2.15 years, which is the IHME national estimate for TB duration in Uganda.

$$E[Duration] = 2.15$$

The results of this alternate model are shown below in Supp. Figure 3, using the same color scheme as manuscript Figure 3. The alternate model estimated that TB incidence ranged from a low of 73 cases per 100,000 in Bukedea District, Eastern Region to a high of 1,139 cases per 100,000 in Kalangala District, Central Region. The 2019 incidence estimates from alternative model are compared with the manuscript estimates in Supp. Figure 4. Compared to the base model, the alternate model yielded incidence estimates that were 16.3% lower on average, but none of these differences were statistically significant.

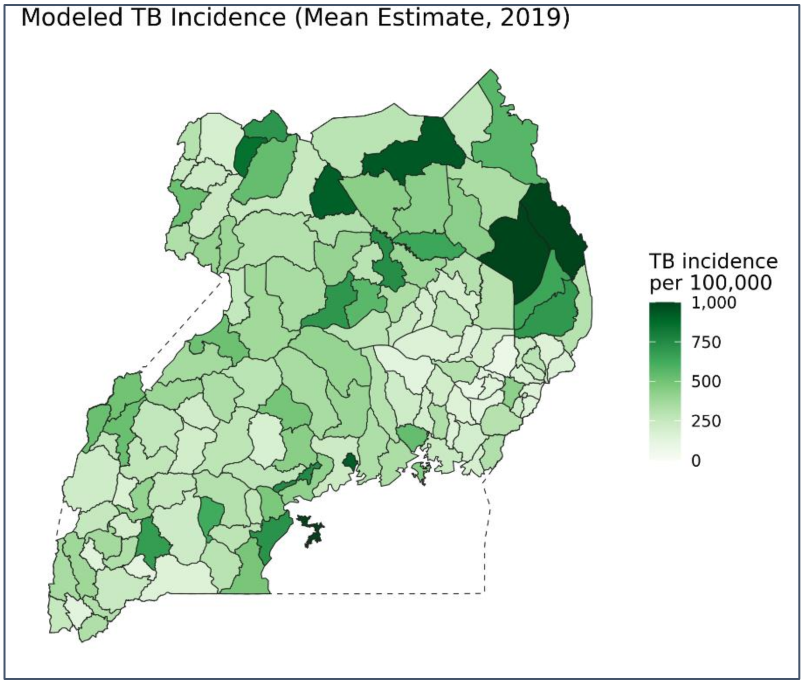

Supp. Figure 3: Estimated incidence of pulmonary TB per 100,000 population by district in Uganda, 2019, using an alternative model with a fixed duration of 2.15 years

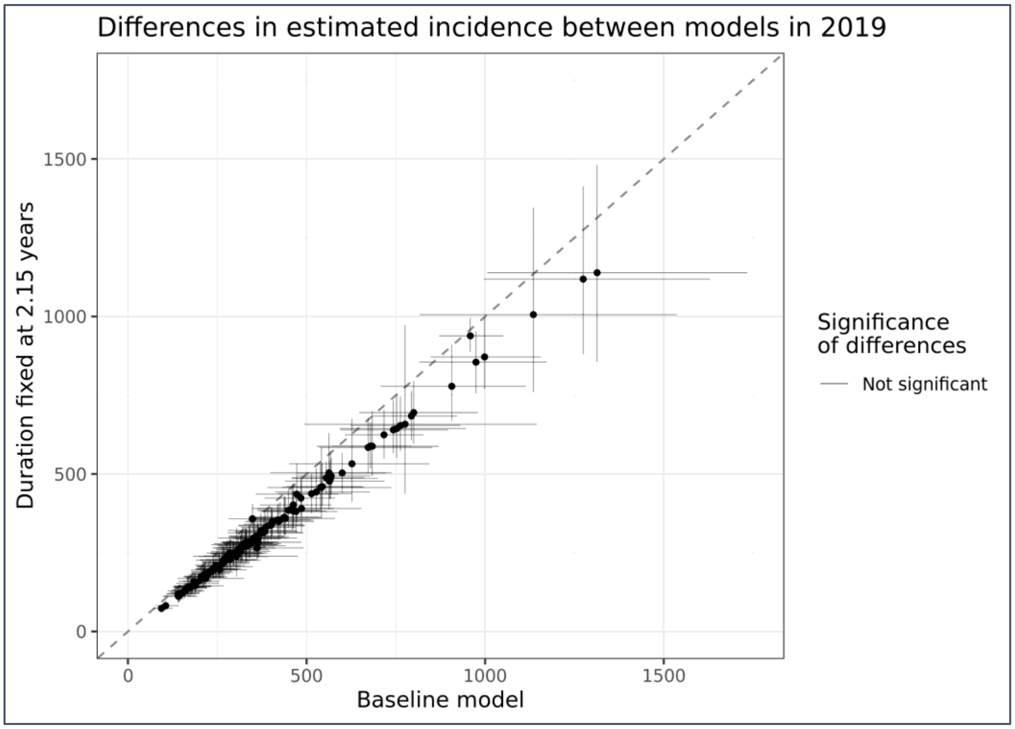

Supp. Figure 4: Scatterplot comparing estimated TB incidence in 2019 between the default model described in the manuscript (X axis) and an alternative model with a fixed duration of 2.15 years (Y axis). 95% uncertainty intervals for both sets of estimates are shown as horizontal and vertical bars.

C. Fixed duration of 1 year

Finally, we conducted a sensitivity analysis using an alternative model where duration of active TB was fixed at 1 year. In our derived function for duration (see Section I, above), expected values for duration can range from 1.51 years to 1.09 years, so a fixed duration of 1 year is relatively low compared to our model estimates.

$$E[Duration] = 1.0$$

The results of this alternate model are shown below in Supp. Figure 5, using the same color scheme as manuscript Figure 3. The alternate model estimated that TB incidence ranged from a low of 117 cases per 100,000 in Bukedea District, Eastern Region to a high of 1,611 cases per 100,000 in Kalangala District, Central Region.

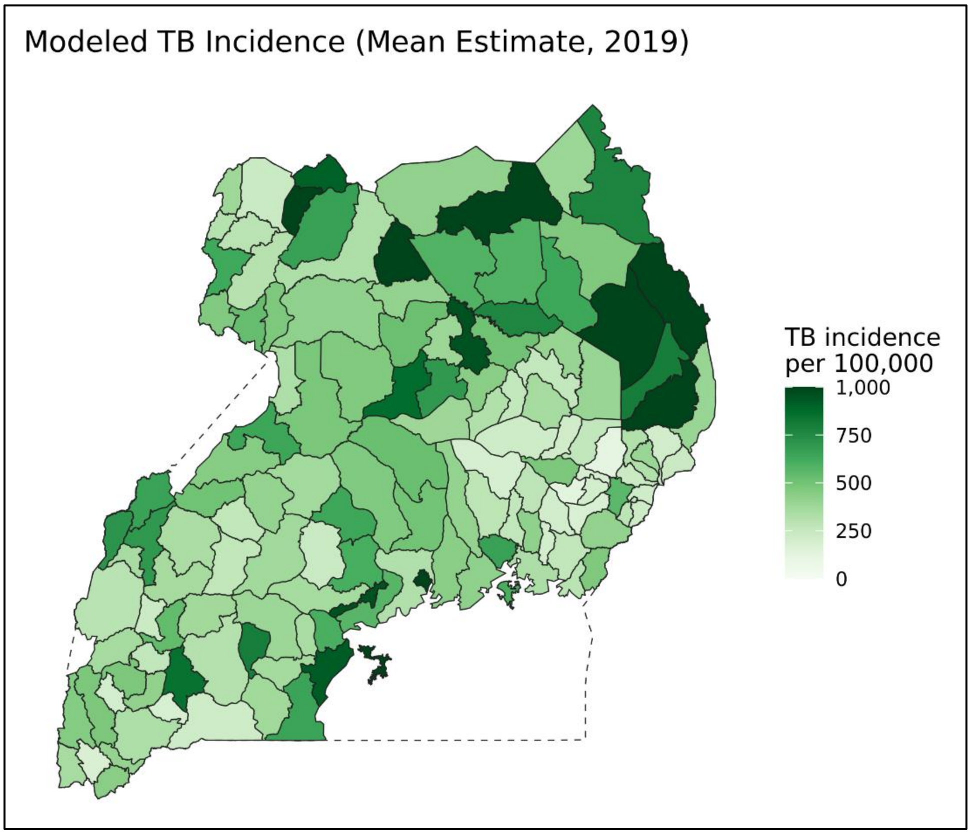

Supp. Figure 5: Estimated incidence of pulmonary TB per 100,000 population by district in Uganda, 2019, using an alternative model with a fixed duration of 1 year

The 2019 incidence estimates from alternative model are compared with the manuscript estimates in Supp. Figure 6. Compared to the base model, the alternate model yielded incidence estimates that were 21.8% higher on average, but none of these differences were statistically significant.

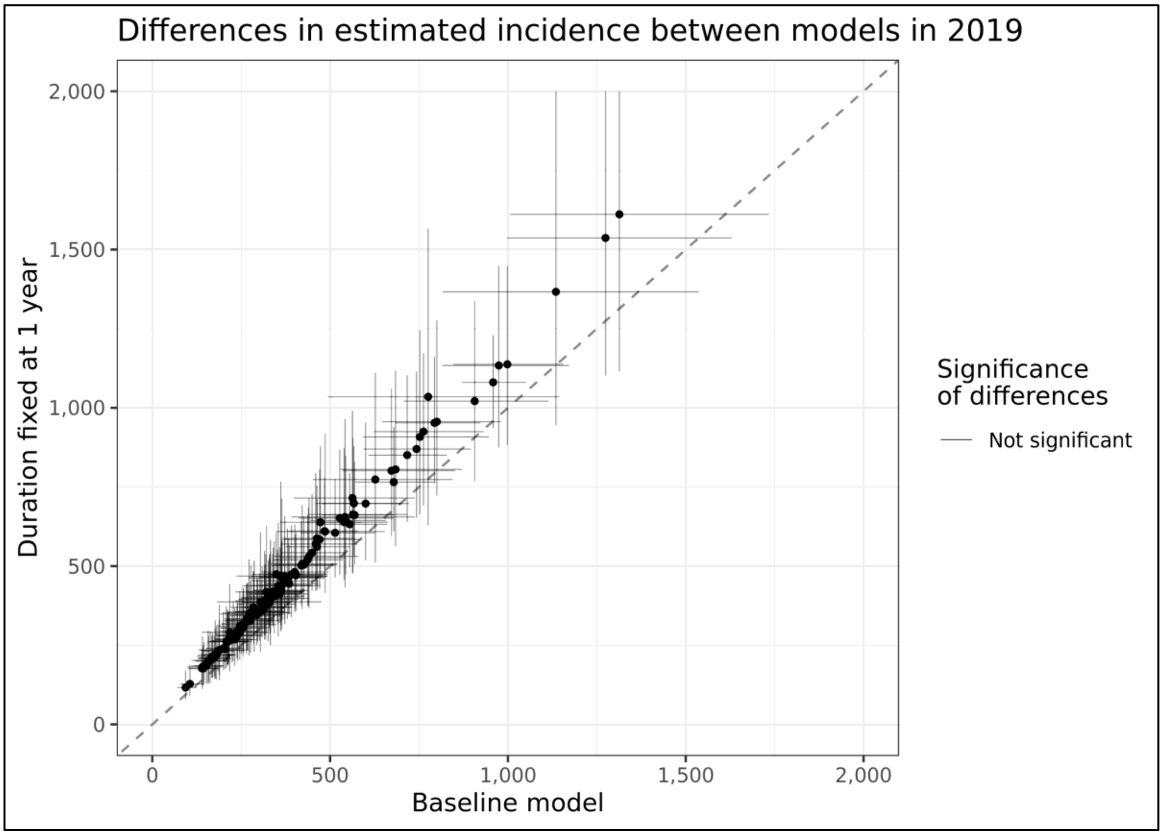

Supp. Figure 6: Scatterplot comparing estimated TB incidence in 2019 between the default model described in the manuscript (X axis) and an alternative model with a fixed duration of 1 year (Y axis). 95% uncertainty intervals for both sets of estimates are shown as horizontal and vertical bars.

V. Out-of-sample comparison to a prevalence-only model

We compare our model to two other approaches that are commonly used for estimating subnational TB burden:

1. *National average model:* estimates TB prevalence for all districts to be equal to the national average
2. *Survey-only model:* a spatial model using only data from a TB prevalence survey plus spatial covariates.

One challenge for validating spatial TB models is that no single data source can be used as a "gold standard" dataset at the subnational level. The unadjusted prevalence survey data should not be considered a "gold standard" at the subnational level, for two reasons. First, because the National TB Prevalence Survey was not powered for subnational estimation, data in some districts suffer from small sample sizes, including districts where no positive TB cases were identified. This sampling variability is accounted for in the modeling framework, but it remains a caveat when using the prevalence survey for validation. Second, in some districts, case notification rates indicate that prevalence should be higher than the district-level estimates from the National TB Prevalence Survey.

We first validate the three candidate models against the unadjusted TB prevalence survey data, then show how the alternative models produce implausible results when compared to case notifications. Finally, we create an adjusted dataset that is plausible given both case notifications and prevalence survey data, and we validate the three candidate models against this adjusted dataset.

A. Validation against prevalence survey data

We performed leave-one-out cross validation on the three candidate models and compared the results. Prevalence observations from the 2014-2015 National TB Prevalence Survey spanned 58 districts: we cross-validated the model by dropping a prevalence observation in one district, training each model type on the remaining data, and comparing predicted prevalence in that district to the dropped data point. These comparisons must be interpreted with caution due to the data limitations described in the previous section.

Supp. Table 4 lists in-sample and out-of-sample predictive metrics for the three candidate models when compared against prevalence survey data. The relationships between out-of-sample prevalence predictions and observed prevalence survey data are also shown in Supp. Figure 7, below, with prevalence survey data points on the X axis and out-of-sample predictions on the Y axis.

Supp. Table 4: In-sample and out-of-sample predictive metrics for the three model alternatives when comparing to unadjusted TB Prevalence Survey data, aggregated by district. RMSE and UI width are both defined in units of prevalent cases per 100,000 population.

**Model**	**In-sample metrics**			**Out-of-sample metrics**		
	RMSE	*R*	Average UI width	RMSE	*R*	Average UI width
National average model	417	*N/A*	*N/A*	423	-0.94	*N/A*
Prevalence-only model	195	0.95	2,549	484	-0.32	1,037
Joint model	406	0.40	279	465	0.22	291

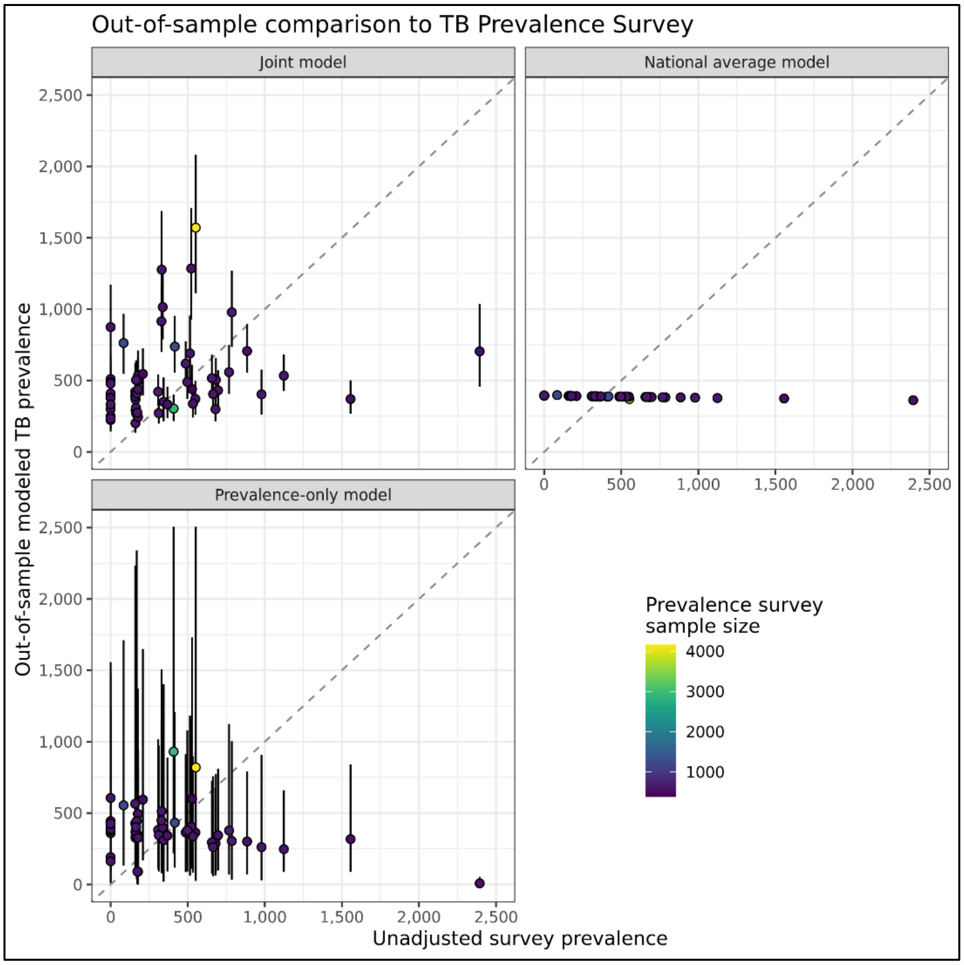

<u>Supp. Figure 7</u>: Scatterplots comparing observed district-level prevalence from the 2014-2015 National TB Prevalence Survey (X axis) with out-of-sample predictions for TB prevalence in those districts (Y axis) for the three candidate models. The X=Y line is shown as a gray dashed line, representing the trend if the out-of-sample models perfectly predicted all prevalence survey data.

Note that the national average model has undefined uncertainty intervals and predicts a constant for the in-sample model, so Pearson's correlation is undefined. In this comparison, the national average model has the lowest out-of-sample RMSE, although the estimates are negatively correlated with actual values (because the held-out data point is excluded when calculating each out-of-sample average). Between the two other models, the survey-only model has much wider uncertainty: the average width of the 95% uncertainty interval is 291 per 100,000 for the joint model, and 1,037 per 100,000 for the survey-only model.

B. Comparison to a prevalence floor

Next, we compare the out-of-sample prevalence estimates from all three models with a "prevalence floor," the lowest prevalence level consistent with observed case notifications in a district. We calculate the prevalence floor by treating case notifications from 2016 through 2019 as complete, which yields the following equation:

$$Prevalence_d^{Floor} = \underset{t}{\text{mean}}\left[\frac{Y_{d,t}^{Notif}}{N_{d,t}^{Notif}}\right] * 1.09$$

In the equation above, $Prevalence_d^{Floor}$ is the lowest prevalence consistent with observed case notifications in each district (d) across the years (t) 2016 to 2019. We estimate prevalence for a district as incidence times duration. If case notifications are complete in a district, then incidence can be estimated as the mean of the annual case notification rate for that district ($Y_{d,t}^{Notif}/N_{d,t}^{Notif}$) across the years 2016 to 2019. Duration is estimated using the formula derived in Supplementary Appendix Section I, with completeness set to 1: $1.51 - 0.42 * 1 = 1.09$. Assuming case notifications are complete yields the lowest TB prevalence consistent with those case notifications; if case notifications are incomplete, then the case notification rate should be scaled up to get true incidence, and the estimated duration would be higher. We should expect a well-performing model to generate prevalence estimates that are higher than the calculated prevalence floor in each district.

The results of this second comparison are shown in Supp. Figure 8 below. The out-of-sample joint model (top left) generated estimates with 95% uncertainty intervals completely above the prevalence floor. By contrast, in the out-of-sample prevalence survey-only model (bottom left), 54 of 58 prevalence estimates had 95% UI bounds that fell partially below the prevalence floor, and mean estimates for 12 districts fell below the prevalence floor. This suggests that in many districts, the out-of-sample TB prevalence estimates generated by the survey-only model are inconsistent with observed case notification counts.

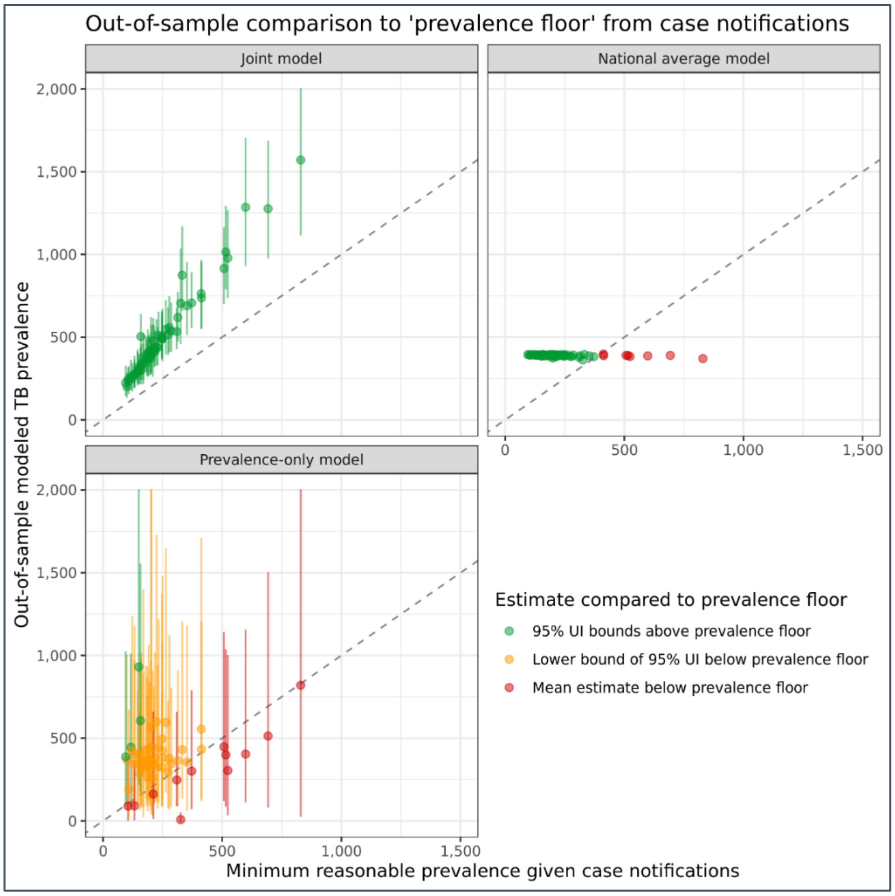

Supp. Figure 8: Scatterplots comparing out-of-sample prevalence predictions from the two spatial models (Y axis) against a "prevalence floor" calculated from case notification rates (X axis). The Y=X line is shown as a gray dashed line; estimates falling below this are inconsistent with observed case notifications.

C. Validation against an adjusted prevalence dataset

To synthesize the two previous validation steps, we constructed a validation dataset that is plausible given both prevalence survey data and case notifications. For the 58 districts that contain prevalence survey sampling clusters, we took either the observed prevalence data or the prevalence floor derived from case notifications, whichever was larger. Supp. Figure 9 shows the adjusted validation dataset compared to the original prevalence survey dataset.

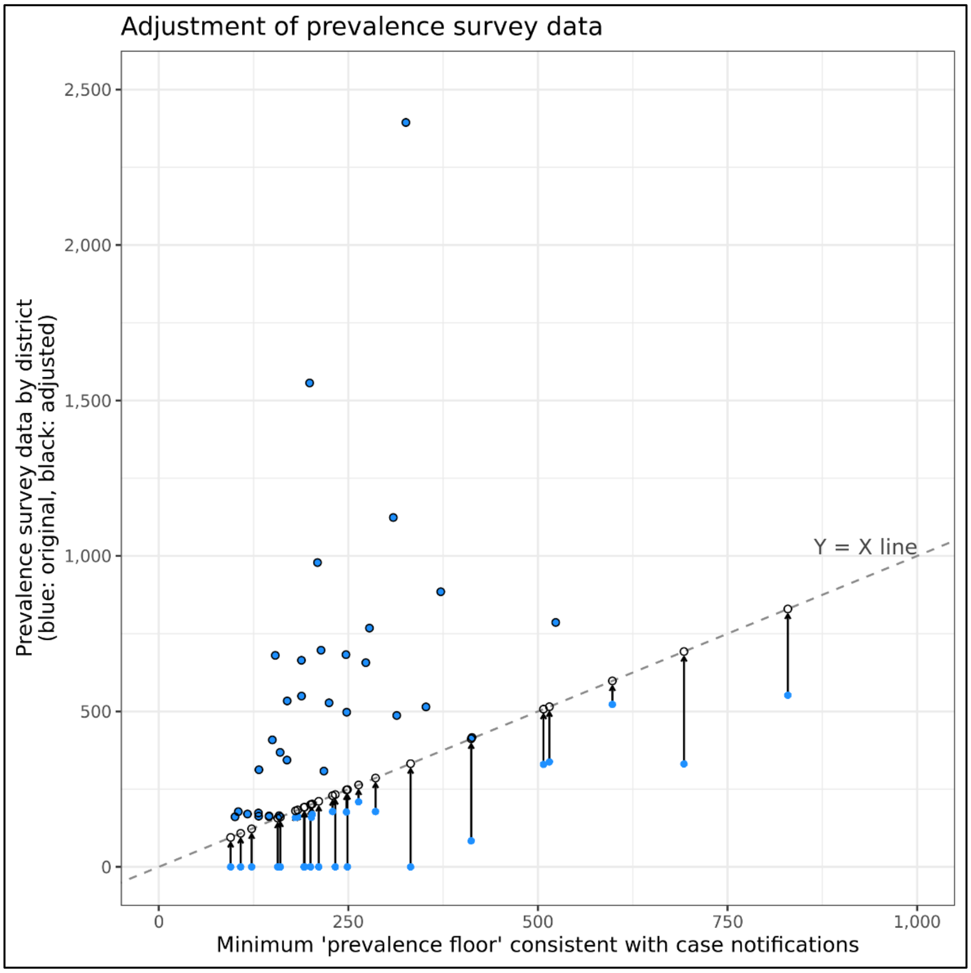

Supp. Figure 9: Scatterplot comparing district-level prevalence estimates from the National TB Prevalence Survey, on the X axis, with the minimum prevalence that would be consistent with observed case notifications, on the Y axis. The original data are shown as solid blue points; the adjusted validation data points are shown in the black point outlines.

Supp. Table 5 lists in-sample and out-of-sample predictive metrics for the three candidate models when compared against this adjusted validation dataset.

Supp. Table 5: In-sample and out-of-sample predictive metrics for the three candidate models when comparing to the adjusted validation dataset. RMSE and UI width are both defined in units of prevalent cases per 100,000 population.

**Model**	**In-sample metrics**			**Out-of-sample metrics**		
	RMSE	*R*	Average UI width	RMSE	*R*	Average UI width
National average model	391	*N/A*	*N/A*	397	-0.92	*N/A*
Prevalence-only model	175	0.95	2,549	453	-0.26	1,037
Joint model	332	0.53	279	391	0.36	291

When evaluated against the adjusted validation dataset that is consistent with both the National TB Prevalence Survey and case notifications, the joint model has the lowest RMSE of the three alternatives; it is also the only model with a positive correlation between out-of-sample predictions and the held-out observations. Based on these validation results, we contend that the joint model presented in this study is the most consistent with both prevalence survey and case notification data.

Because case notifications are understood to be incomplete in many districts, the true prevalence corresponding to case notification rates is higher than the prevalence floor. Using a different prevalence adjustment that accounts for incomplete case notifications would likely further improve validation metrics for the joint model, although such a validation would also risk over-reliance on model assumptions.

VI. References

1. Freeman, J. & Hutchison, G. B. Prevalence, incidence, and duration. *Am J Epidemiol* **112**, 707–723 (1980).

2. World Health Organization. *Guidelines for Treatment of Drug-Susceptible Tuberculosis and Patient Care*. (World Health Organization, Geneva, 2017).

3. Tiemersma, E. W., van der Werf, M. J., Borgdorff, M. W., Williams, B. G. & Nagelkerke, N. J. D. Natural history of tuberculosis: Duration and fatality of untreated pulmonary tuberculosis in HIV negative patients: A systematic review. *PLoS One* **6**, (2011).

4. Payne, B. & Bellamy, R. Managing tuberculosis in people with HIV. *BMJ Clin Evid* (2007).

5. Falzon, D. *et al.* WHO guidelines for the programmatic management of drug-resistant tuberculosis: 2011 update. *European Respiratory Journal* **38**, 516–528 (2011).

6. World Health Organization. *WHO Consolidated Guidelines on Tuberculosis. Module 4: Drug-Resistant Tuberculosis Treatment*. https://www.who.int/publications/i/item/9789240007048 (2020).

7. Bastard, M. *et al. Methods Used by WHO to Estimate the Global Burden of TB Disease: Global Tuberculosis Report 2023*. https://www.who.int/publications/m/item/methods-used-by-who-to-estimate-the-global-burden-of-tb-disease-2023 (2023).

8. Buregyeya, E., Criel, B., Nuwaha, F. & Colebunders, R. Delays in diagnosis and treatment of pulmonary tuberculosis in Wakiso and Mukono districts, Uganda. *BMC Public Health* **14**, 1–10 (2014).

9. Dwyer-Lindgren, L. *et al.* Mapping HIV prevalence in sub-Saharan Africa between 2000 and 2017. *Nature* **570**, 189–193 (2019).

10. Ledesma, J. R. *et al.* Global, regional, and national sex differences in the global burden of tuberculosis by HIV status, 1990–2019: results from the Global Burden of Disease Study 2019. *Lancet Infect Dis* **3099**, 1–20 (2021).

11. Uganda National Tuberculosis and Leprosy Program. *National Tuberculosis and Leprosy Division: July 2017-June 2018 Report*. (2018).

12. Uganda Ministry of Health. *The Uganda National Tuberculosis Prevalence Survey, 2014-2015: Survey Report*. https://www.health.go.ug/cause/the-uganda-national-tuberculosis-prevalence-survey-2014-2015-survey-report/ (2015).

13. Glaziou, P., Dodd, P. J., Dean, A. S. & Floyd, K. *Methods Used by WHO to Estimate the Global Burden of TB Disease: Global Tuberculosis Report 2019*. https://www.who.int/docs/default-source/documents/tuberculosis/technical-appendix-global-tb-report-2019methods-used-by-who-to-estimate-the-global-burden-of-tb-disease.pdf?sfvrsn=e9c1d7b0_2 (2019).

14. Law, I. *et al.* National tuberculosis prevalence surveys in Africa, 2008–2016: an overview of results and lessons learned. *Tropical Medicine and International Health* **25**, 1308–1327 (2020).

15. Simpson, D., Rue, H., Riebler, A., Martins, T. G. & Sørbye, S. H. Penalising model component complexity: A principled, practical approach to constructing priors. *Statistical Science* **32**, 1–28 (2017).

16. Tatem, A. J. WorldPop, open data for spatial demography. *Sci Data* **4**, 2–5 (2017).

17. Phan, D. H. Lights and GDP relationship: What does the computer tell us? *Empir Econ* **65**, 1215–1252 (2023).

18. Thompson, C. G., Kim, R. S., Aloe, A. M. & Becker, B. J. Extracting the Variance In flation Factor and Other Multicollinearity Diagnostics from Typical Regression Results. *Basic Appl Soc Psych* **39**, 81–90 (2017).

19. Fullman, N. *et al.* Measuring performance on the Healthcare Access and Quality Index for 195 countries and territories and selected subnational locations: a systematic analysis from the Global Burden of Disease Study 2016. *The Lancet* **391**, 2236–2271 (2018).
